# Combination therapy using Pentostam and Praziquantel improves lesion healing and parasite resolution in BALB/c mice co-infected with *Leishmania major* and *Schistosoma mansoni*

**DOI:** 10.1186/1756-3305-6-244

**Published:** 2013-08-22

**Authors:** Christopher Khayeka–Wandabwa, Helen Lydiah Kutima, Venny CS Nyambati, Johnstone Ingonga, Elijah Oyoo–Okoth, Lucy Wanja Karani, Bernard Jumba, Kiige Samuel Githuku, Christopher O Anjili

**Affiliations:** 1 Institute of Tropical Medicine and Infectious Diseases (ITROMID), Jomo Kenyatta University of Agriculture and Technology (JKUAT), P.O. Box 62000-00200, Nairobi, Kenya; 2 Biochemistry and Zoology departments, Jomo Kenyatta University of Agriculture and Technology (JKUAT), P O Box 62000 00200, Nairobi, Kenya; 3 Centre for Biotechnology Research and Development (CBRD), Kenya Medical Research Institute (KEMRI), P O Box 54840 00200, Nairobi, Kenya; 4 Division of Environmental Health, School of Environmental Studies, University of Eldoret, P O Box 1125 30100, Eldoret, Kenya; 5 School Natural Resources and Environmental Studies, Karatina University, P O Box 1957 10101, Karatina, Kenya; 6 Department of Biological Science (Parasitology), University of Eldoret, P O Box 1125 30100, Eldoret, Kenya; 7 Department of Zoological Sciences, Kenyatta University, P O Box 43844 00100, Nairobi, Kenya

**Keywords:** Schistosomiasis, Leishmaniasis, *Leishmania major*, *Schistosoma mansoni*, Praziquantel, Drug efficacy, Pentostam, Co-infection, Combination therapy

## Abstract

**Background:**

Most natural host populations are exposed to a diversity of parasite communities and co-infection of hosts by multiple parasites is commonplace across a diverse range of systems. Co-infection with *Leishmania major* and *Schistosoma mansoni* may have important consequences for disease development, severity and transmission dynamics. Pentavalent antimonials and Praziquantel (PZQ) have been relied upon as a first line of treatment for *Leishmania* and *Schistosoma* infections respectively. However, it is not clear how combined therapy with the standard drugs will affect the host and parasite burden in concomitance. The aim of the current study was to determine the efficacy of combined chemotherapy using Pentostam and PZQ in BALB/c mice co-infected with *L. major* and *S. mansoni*.

**Methods:**

The study used BALB/c mice infected with *L. major* and *S. mansoni*. A 3 × 4 factorial design with three parasite infection groups (Lm, Sm, Lm + Sm designated as groups infected with *L. major*, *S. mansoni* and *L. major* + *S. mansoni,* respectively) and four treatment regimens [P, PZQ, P + PZQ and PBS designating Pentostam®(GlaxoSmithKline UK), Praziquantel (Biltricide®, Bayer Ag. Leverkusen, Germany), Pentostam + Praziquantel and Phosphate buffered saline] as factors was applied. In each treatment group, there were 10 mice. Lesion development was monitored for 10 weeks. The parasite load, body weight, weight of the spleen and liver were determined between week 8 and week 10.

**Results:**

Chemotherapy using the first line of treatment for *L. major* and *S. mansoni* reduced the lesion size and parasite loads but did not affect the growth response, spleen and liver. In the co-infected BALB/c mice, the use of Pentostam or PZQ did not result in any appreciable disease management. However, treatment with P + PZQ resulted in significantly (*p* < 0.05) larger reduction of lesions, net increase in the body weight, no changes in the spleen and liver weight and reduced Leishman-Donovan Units (LDU) and worm counts than BALB/c mice treated with Pentostam or PZQ alone.

**Conclusions:**

The present study demonstrated that the combined first line of treatment is a more effective strategy in managing co-infection of *L. major* and *S. mansoni* in BALB/c mice.

## Background

Most natural host populations are exposed to a diverse community of parasites, and with respect to epidemiologic overlaps and human economic dynamics, comorbidity of hosts by multiple parasites occurs across a diverse range of systems [1-4]. Recent studies in vertebrates suggest that interactions between co-infecting parasites have important consequences for disease development, severity and transmission dynamics [5,6]. Thus understanding the consequences of co-infections in a host may be crucial for a deeper understanding of the disease pathognomonic, epidemiology and effective disease control strategies.

In the developing world, leishmaniasis, caused by obligate intracellular kinetoplastid protozoa of the genus *Leishmania*, is endemic [7,8] and schistosomiasis, caused by parasitic trematodes (schistosomes) have widely been reported [9-13]. *Leishmania* and *Schistosoma* overlap extensively in their epidemiological distributions and occasionally co-infect the same individuals [14-21]. How these two types of parasite might interact within co-infected hosts and the associated epidemiology continues to be debated. One line of argument indicates that the interactions between the helminth and protozoan parasites could affect both parties [22], while others argue that the helminth/protozoan co-infection influences leishmaniasis development without any effect on the helminth parasite [23-25]. This bias may reflect the greater human disease burden imposed by leishmaniasis compared to helminths [26], as well as the ongoing need to understand what causes variability in leishmaniasis infection outcomes.

Interactions among parasitic agents commonly alter disease severity [1,27,28]. Co-infecting parasites may interact either positively (facilitation) or negatively (competition) via a range of mechanisms including resource competition, immune-mediated interactions and direct interference. To date, studies of helminth-protozoa co-infection have focused largely on immune-mediated mechanisms, no doubt largely due to the known immunomodulatory effects of helminths. Using the immune response mechanism, two major pathways have been proposed by which helminths might release parasites from immune pressure and thereby facilitate their replication, both of which involve the dampening of pro-inflammatory immune responses [29-34]. Thus it has been suggested that by polarizing immune responses towards Th2-type effector mechanisms, helminths will diminish the pro-inflammatory Th1-type mechanisms needed to down modulate *Leishmania* in comorbidity. These suggest that helminth co-infection might thus impair the mechanisms necessary to control leishmaniasis. The current immunomodulatory explanation is in concurrence with observations in murine models, where co-infection of *L. major* and *S. mansoni* exacerbated lesion development compared to mice infected with *L. major* alone [22-24,35]. The action of helminth infection affecting the immune response of the host, may increase protozoan multiplication substantially thus enhancing leishmaniasis severity [23,24,26].

The chemotherapy of leishmaniasis is based on the use of antimoniates, among which the most used are meglumine antimoniate (Glucantime®) and sodium stibogluconate (Pentostam®) [36-38]. The WHO recommended schistosomiasis control strategies for humans by focusing on the large-scale population-based and repeated chemotherapy, which is still the key strategy today. Three drugs have been used, which differ in their effects on schistosome species: metrifonate (targeting *S. haematobium*), oxamniquine (targeting *S. mansoni*), and PZQ (for all human species) [39,40]. Due to its broader spectrum, PZQ has finally become the first-line medicine. Yet, advances in antiparasitic chemotherapy have made combination chemotherapy a real possibility [41,42]. Drug combinations aim to delay or prevent the emergence of resistance, shorten the course of treatment and lower required doses. Other potential advances include convenience, better compliance and lower costs [43]. Nevertheless, the co-infection may potentially affect the chemotherapy outcome using standard and/or recommended drugs for single infections in the mice. As yet, the pathognomonic and subsequent treatment of protozoa and helminth concomitance in murine models is not well understood. The aim of the current study was to determine the efficacy of combined chemotherapy using Pentostam® and Praziquantel in BALB/c mice co-infected with *L. major* and *S. mansoni*. We hypothesize that treatment of BALB/c mice co-infected with *L. major* and *S. mansoni* using a combination of Pentostam and PZQ as the first line treatments *for L. major* and *S. mansoni* respectively may result in synergistic drug actions against the infection.

## Methods

### Experimental setup

The study applied a 3 × 4 factorial design with three parasite infection groups (Lm, Sm and Lm + Sm designated as groups infected with *L. major*, *S. mansoni* and *L. major* + *S. mansoni* respectively) and four treatment regimens [P, PZQ, P + PZQ and PBS designating Pentostam®(GlaxoSmithKline, UK), Praziquantel(Biltricide®, Bayer Ag. Leverkusen, Germany), Pentostam + Praziquntel and Phosphate buffered saline] as factors. In each treatment group, there were 10 mice. All treatments were executed in triplicate.

### Mice, parasites and experimental infections

Female (6–8 week old) BALB/c mice weighing 20 ± 2 g were used in the experiment. The animals were obtained from Kenya Medical Research Institute (KEMRI) animal breeding facility, Nairobi-Kenya. The animals were moved into the experimental room for acclimatization one week before the start of the experiments. The mice were housed in 15 cm × 21 cm × 29 cm transparent plastic cages. They were fed with pellets (Mice pellets UNGA® feeds) and water *ad libitum*.

Kenya laboratory maintained (KEN-lab) strain of *S. mansoni* parasite was used in the study. The isolate had been routinely maintained in the laboratory at KEMRI by passage in *Biomphalaria pfeifferi* snails and inbred laboratory mice. For mouse infections, cercariae were obtained from infected snails, counted and applied percutaneously by the ring method [44] to mice anesthetized intraperitoneally with Pentobarbital sodium 80 mg/kg (Rompun; Bayer Plc., Newbury, UK) [45]. For the *S. mansoni* infection, mice were infected with 70 cercariae.

*L. major* (strain IDUB/KE/94 = NLB-144) was maintained by serial passage in BALB/c mice to maintain virulence. An aspirate isolate from the footpad of an infected BALB/c mouse was cultivated in Schneider’s *Drosophila* insect medium (Sigma, Saint Louis, USA), supplemented with 20% heat inactivated foetal bovine serum (FBS) (Cultilab, Campinas, SP, Brazil), 500 μg/ml penicillin, 500 μg/ml streptomycin and 250 μg/ml 5-fluorocytosine arabinoside (all from Gibco, Grand Island, NY, USA) [46,47]. Promastigotes were incubated at 25°C and grown to stationary phase to generate infective metacyclic forms at 6th day of culture. Promastigotes in the medium were counted with a hemocytometer (Improved Double Neubauer) (Pharmacia-GE Healthcare, Uppsala, Sweden) with a Nikon optiphot optical microscope at 40× magnification. Mice were inoculated with 1 × 10^6^ stationary phase *L. major* promastigotes in 50 μl PBS into the Left Hind Footpad (LHFP) using a 29 gauge needle.

### Treatment

At the 5th week post infection (day 29), each of the parasite infection groups, were either treated with Pentostam (P), Praziquantel (PZQ), P + PZQ or PBS for the controls. 20 mg/kg bodyweight/day standard dose of Pentostam (GlaxoSmithKline, UK), was prepared and administered intraperitoneally (ip) for 28 days [48]. Praziquantel (Biltricide®, Bayer Ag. Leverkusen, Germany) was suspended in PBS and was administered to the mice by gavage at a total dosage of 600 mg/kg body weight (divided into 2 equal doses of 300 mg/kg) given 8 hours apart [49,50].

### Sampling, lesion measurement and determination of parasite burden

A total of five mice were sampled at week 8 and week 10 for analysis of *L. major* parasite loads and *S. mansoni* worm counts. Lesion sizes of *L. major* infected mice, which was defined as the difference in thickness between the infected footpad and the non-infected contralateral footpad, was monitored weekly by measurement using a Starret dial caliper (Mitutoyo, Suzano, SP, Brazil) [24,51]. The weight of the mice was also monitored on a weekly basis.

*S. mansoni* worms were recovered by portal perfusion [52], with perfusion buffer [phosphate buffered saline, 0.02 μl/ml heparin (monoparin; CP Pharmaceuticals Ltd.,Wrexham, UK)]. The worms were washed free of erythrocytes and counted using a dissecting microscope. The percentage worm recovery and percentage worm reduction was calculated [50] as shown below:

$$\begin{array}{l} \%\text{worm recovery}\\ \;=\frac{\text{Mean of total worms in Experimental group}}{\text{Mean of worms in infected control}}*100 \end{array}$$

$$\%\text{worm}\;\text{reduction}=\frac{\begin{array}{l} \;\text{Mean of worms in Infected Control}‒\\ \text{Mean of worms in Experimental group} \end{array}}{\text{Mean of worms in infected control}}*100$$

The liver and spleen were removed and weighed and changes post-infection and chemotherapy were determined based on percentage reduction/increase, spleeno-somatic and hepato-somatic indices [50]. Splenic *L. major* burdens were determined from Giemsa-stained impression smears and expressed as Leishman-Donovan units (the number of amastigotes per 1000 host nuclei, multiplied by the weight of the organ) [53-55].

### Ethical clearance

All procedures were approved by the ethics committees for animal care and research: KEMRI Animal Care and Use Committee (ACUC), Scientific Steering Committee (SSC) and Ethical Review Committee (ERC). The guidelines were strictly adhered to during the research.

### Statistical analysis

All statistical analyses were performed with a version of STATISTICA 10.0 statistical packages. Normality of data distributions were checked by means of the kurtosis to determine any need for applying appropriate data transformation procedures [55]. For each parameter analyzed, differences among treatment groups exposed to different drugs were tested by Factorial ANOVA. Comparison of efficacies among different treatment groups and time was carried out using factorial repeated measure ANOVA. Differences on *L. major* parasites and *S. mansoni* worm counts were analyzed using Friedman’s Two-Way ANOVA as count data take discreet variables. Percentage data were arcsine transformed before subjecting the data to statistical analysis of Variance. In cases where significant differences were discerned means were compared using Tukey’s HSD test [56]. All analyzed results were declared significant at *p* < 0.05.

## Results

**Figure 1:**
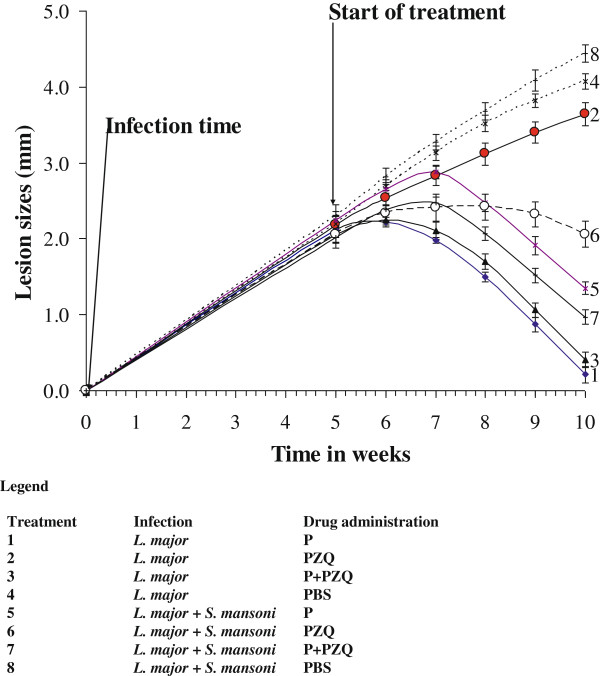
Lesion sizes of BALB/c mice at the start of infection, during the start of treatment with Pentostam, PZQ and combination of Pentostam and PZQ for 10 weeks.

The lesion sizes of BALB/c mice at the start of infection, at treatment and response after treatment with Pentostam, PZQ and combination of P + PZQ between week 8 and 10 is shown in Figure 1. During the first five weeks, there was a progressive increase of lesion size in BALB/c mice indicating progression of disease infection. There were significant differences in lesion sizes among different treatments (F = 21.1223, *p* = 0.0001) between week 5 to week 10. The largest reduction in lesion size after five weeks post infection followed by chemotherapy was observed in *L. major* infected BALB/c mice treated with Pentostam. Nevertheless, there was no (*p* > 0.05) difference in lesion size of *L. major* infected BALB/c mice treated with either Pentostam or with a combination of P + PZQ. In the co-infected BALB/c mice, treatment with P + PZQ resulted in significantly (*p* < 0.05) the largest reduction of lesion when compared with BALB/c mice treated with Pentostam or PZQ alone. PZQ had no effect on *L. major* infected BALB/c mice.

Body weights, weights of spleen and weights of the liver of BALB/c mice infected with *L. major*, *S. mansoni* and those co-infected with *L. major* + *S. mansoni* are presented in Table 1. There were significant differences in the body weights, weight of spleen and in the weight of liver of BALB/c mice under different treatments between the eighth and tenth week (*p* < 0.05). The largest increase in body weight occurred in mice infected with *L. major* and treated with Pentostam, in *S. mansoni* infected mice and treated with PZQ; nonetheless, combined therapy resulted in net increase in body weights of BALB/c mice singly infected with either *L. major* and *S. mansoni*. Treatment of *L. major* and *S. mansoni* co-infected BALB/c mice through monotherapy (either Pentostam or PZQ) resulted in reduced body weights while using combination therapy of P + PZQ resulted in a net increase in the body weight between week 8 and 10. It was observed that the largest increase in the weight of the spleen was in control groups. As for the infected and treated mice, in the *L. major* or *S. mansoni* infected mice, treatment with Pentostam and PZQ respectively, resulted in a slight and non-significant (*p* > 0.05) increase in the weight of spleen between weeks 8 and 10. Simultaneous chemotherapy for either infection did not show any appreciable change in the weight of spleen. However, the largest increase in spleen weight occurred in BALB/c infected with *L. major* and treated with PZQ, and in BALB/c infected with *S. mansoni* and treated with Pentostam. In the co-infected mice, treatment with Pentostam or PZQ alone resulted in a significant (*p* < 0.05) increase in the weight of spleen between weeks 8 and 10. On the contrary, treatment of BALB/c co-infected with *L. major* + *S. mansoni* with P + PZQ resulted in slight but non-significant (*p* > 0.05) increase in weight of the spleen. Treatment of BALB/c mice infected with *L. major* or *S. mansoni* with Pentostam and PZQ respectively or a combination of P + PZQ resulted in a slight reduction in the weight of the liver. There were opposing trends in the weight of the liver when treatment of *L. major* or *S. mansoni* infection was conducted using PZQ and Pentostam respectively. In mice, co-infected with *L. major* + *S. mansoni* there were significant (*p* < 0.05) increases in the weight of the liver when Pentostam and PZQ were used as respective chemotherapeutants. Nevertheless, in co-infected mice, treatment with combined therapy of P + PZQ resulted in a slight but non-significant (*p* > 0.05) increase in the liver weight. All the controls resulted in significant (*p* < 0.05) increases in weight of the liver.

**Table 1:** **Mean (± SEM) body weight, weight of spleen and weight of the liver of BALB/c mice infected with *L. major*, *S. mansoni *and those co-infected with *L. major*** **+** ***S. mansoni***

**Parasite infection**		**Body weights (g)**			**Weight of spleen (g)**			**Weight of the liver (g)**		
	**Treatments**	**Week 8**	**Week 10**	**% change**	**Week 8**	**Week 10**	**% change**	**Week 8**	**Week 10**	**% change**
*L. major*	P	18.2 ± 0.8	20.4 ± 1.4	12.1	0.14 ± 0.007	0.15 ± 0.012	7.14	0.81 ± 0.091	0.80 ± 0.084	−1.26
	PZQ	18.0 ± 0.5	16.6 ± 1.4	−7.77	0.20 ± 0.027	0.24 ± 0.009	20.00	0.84 ± 0.014	0.86 ± 0.025	2.13
	P + PZQ	18.6 ± 0.5	20.2 ± 1.2	8.60	0.15 ± 0.005	0.15 ± 0.007	0.00	0.79 ± 0.015	0.77 ± 0.014	−1.50
	PBS	18.4 ± 0.4	14.4 ± 1.1	−21.7	0.19 ± 0.027	0.24 ± 0.008	26.32	0.84 ± 0.011	0.86 ± 0.014	2.15
*S. mansoni*	P	18.0 ± 0.6	16.1 ± 1.1	−10.6	0.20 ± 0.010	0.24 ± 0.009	20.00	1.59 ± 0.023	1.64 ± 0.012	3.14
	PZQ	18.0 ± 0.6	19.8 ± 0.9	10.0	0.17 ± 0.022	0.19 ± 0.011	11.76	0.83 ± 0.006	0.82 ± 0.004	−1.26
	P + PZQ	18.6 ± 0.8	20.3 ± 0.6	9.10	0.16 ± 0.006	0.16 ± 0.005	0.00	0.83 ± 0.013	0.82 ± 0.049	−1.38
	PBS	18.4 ± 0.9	14.5 ± 1.7	−21.2	0.20 ± 0.025	0.25 ± 0.006	25.00	1.08 ± 0.011	1.11 ± 0.009	2.77
*L. major* + *S. mansoni*	P	19.8 ± 0.2	18.2 ± 0.5	−8.08	0.22 ± 0.006	0.25 ± 0.013	13.60	1.55 ± 0.041	1.58 ± 0.032	3.20
	PZQ	18.4 ± 0.3	17.0 ± 0.8	−7.60	0.22 ± 0.009	0.24 ± 0.007	9.09	1.59 ± 0.012	1.61 ± 0.011	1.95
	P + PZQ	18.4 ± 0.6	19.8 ± 0.4	7.60	0.19 ± 0.016	0.20 ± 0.012	5.26	0.82 ± 0.012	0.83 ± 0.013	1.22
	PBS	17.9 ± 0.9	14.4 ± 1.5	−19.6	0.21 ± 0.007	0.27 ± 0.018	28.57	1.04 ± 0.014	1.08 ± 0.017	4.24
ANOVA	**Main effect**									
	F	19.311	7.421		9.241	8.421		7.112	14.0214	
	*p* value	0.0000	0.0005		0.0001	0.0004		0.0071	0.0000	
	**Repeated measure**									
	F		9.2114			7.5515			5.5544	
	*p* value		0.0007			0.0021			0.0081	

The spleeno-somatic and hepato-somatic indices of BALB/c infected mice under different treatments are shown Table 2. The spleeno-somatic index and hepato-somatic index of *L. major* infected BALB/c mice reduced significantly (*p* < 0.05) between week 8 and week 10 when treatment was carried out using Pentostam and P + PZQ but increased with treatment using PZQ. In BALB/c mice infected with *S. mansoni*, treatment with Pentostam resulted in a significant (*p* < 0.05) increase of spleeno-somatic and hepato-somatic indices. Meanwhile treatment of *S. mansoni* infected BALB/c mice with PZQ resulted in a non-significant (*p* > 0.05) increase in the spleeno-somatic index but significantly reduced the hepato-somatic index. Combined therapy of P + PZQ significantly (*p* < 0.05) reduced the spleeno-somatic and hepato-somatic indices. In co-infected BALB/c mice, treatment with either Pentostam or PZQ resulted in a significant (*p* < 0.05) increase in both the spleeno-somatic and hepato-somatic indices. However, when treatment was done using combined therapy of P + PZQ, the spleeno-somatic index decreased slightly but with a larger significant (*p* < 0.05) reduction observed in hepato-somatic index. Regardless of the parasitic levels of infection of the BALB/c mice, all mice maintained in the control groups had increased spleeno-somatic and hepato-somatic index between week 8 and 10.

**Table 2:** **The spleeno-somatic and hepato-somatic indices of BALB/c infected with *L. major*, *S. mansoni *and those co-infected with *L. major*** **+** ***S. mansoni *undergoing various treatments with Pentostam and PZQ between week 8 and week 10**

**Infection**	**Drug administration**	**Spleeno-somatic index**		**Hepato-somatic index**	
*L. major*		Week 8	Week 10	Week 8	Week 10
	P	0.77 ± 0.031	0.74 ± 0.039	4.45 ± 0.14	3.92 ± 0.08
	PZQ	1.11 ± 0.173	1.45 ± 0.100	4.67 ± 0.15	5.18 ± 0.23
	P + PZQ	0.81 ± 0.016	0.74 ± 0.029	4.25 ± 0.18	3.81 ± 0.13
	PBS	1.03 ± 0.150	1.67 ± 0.084	4.57 ± 0.10	5.97 ± 0.32
*S. mansoni*	P	1.11 ± 0.088	1.49 ± 0.066	8.83 ± 1.11	10.19 ± 0.50
	PZQ	0.94 ± 0.073	0.96 ± 0.064	4.61 ± 0.09	4.14 ± 0.21
	P + PZQ	0.86 ± 0.081	0.79 ± 0.044	4.46 ± 0.07	4.04 ± 0.22
	PBS	1.09 ± 0.081	1.72 ± 0.073	5.87 ± 1.12	7.66 ± 0.27
*L. major*+	P	1.11 ± 0.165	1.37 ± 0.076	7.83 ± 1.44	8.68 ± 2.04
*S. mansoni*	PZQ	1.20 ± 0.111	1.41 ± 0.077	8.64 ± 0.12	9.47 ± 0.13
	P + PZQ	1.03 ± 0.075	1.01 ± 0.041	4.46 ± 0.11	4.19 ± 0.07
	PBS	1.17 ± 0.046	1.88 ± 0.051	5.81 ± 0.16	7.50 ± 0.43

**Figure 2:**
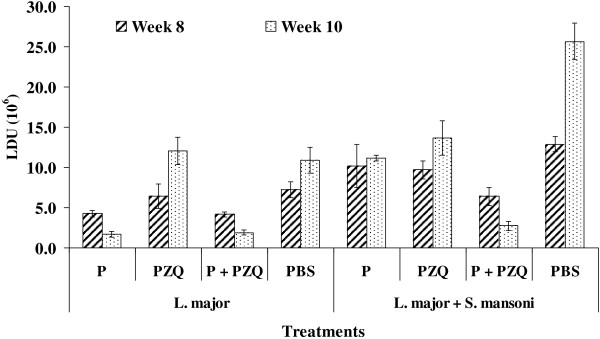
**The LDU of *L. major *parasite in BALB/c mice infected with *L. major *and those co-infected with *L. major *and *S. mansoni *receiving various treatments of Pentostam and PZQ between week 8 and week 10.**

In *L. major* infected BALB/c mice, treatment with Pentostam and P + PZQ resulted in the lowest LDU, which significantly (*p* < 0.05) reduced between week 8 and week 10 (Figure 2). In the PZQ treated and control groups, there was a significant increase in the LDU between week 8 and week 10. In BALB/c mice co-infected with *L. major* + *S. mansoni*, treatment with P + PZQ reduced LDU significantly (*p* < 0.05). In the rest of the treatments there were significant (*p* < 0.05) increase in LDU over the two weeks time.

Trends in the worm counts between *S. mansoni* infected and those co-infected with *L. major* + *S. mansoni* were similar (Table 3). In the Pentostam treated and control groups, there was a significant (*p* < 0.05) increase in the worm counts between week 8 and week 10. In *S. mansoni* infected BALB/c mice, treatment with PZQ and P + PZQ resulted in reduced worm counts. In *L. major* + *S. mansoni* co-infected BALB/c mice, treatment with P + PZQ resulted in a significant (*p* < 0.05) reduction in worm counts as compared to mice treated with PZQ alone.

**Table 3:** **Worm counts of BALB/c infected with *S. mansoni *and those co-infected with *L. major*** **+** ***S. mansoni *under various treatments with Pentostam and PZQ between week 8 and week 10**

**Infection**	**Drug administration**	**Week 8**	**Week 10**
*S. mansoni*	P	34.80 ± 6.09	41.2 ± 2.58
	PZQ	10.4 ± 1.29	8.4 ± 1.50
	P + PZQ	14.4 ± 1.24	11.60 ± 1.24
	PBS	36.20 ± 5.17	46 ± 2.79
*L. major* + *S. mansoni*	P	30.60 ± 7.59	38.6 ± 2.68
	PZQ	11.40 ± 2.74	8.80 ± 1.44
	P + PZQ	7.40 ± 2.60	5.0 ± 1.20
	PBS	34.20 ± 3.14	44.2 ± 2.37
**Friedman Two-Way ANOVA**	**Main effect**		
	F	11.231	19.258
	P-value	0.0002	0.0000
	**Repeated measure**		
	F		7.511
	P-value		0.0014

**Figure 3:**
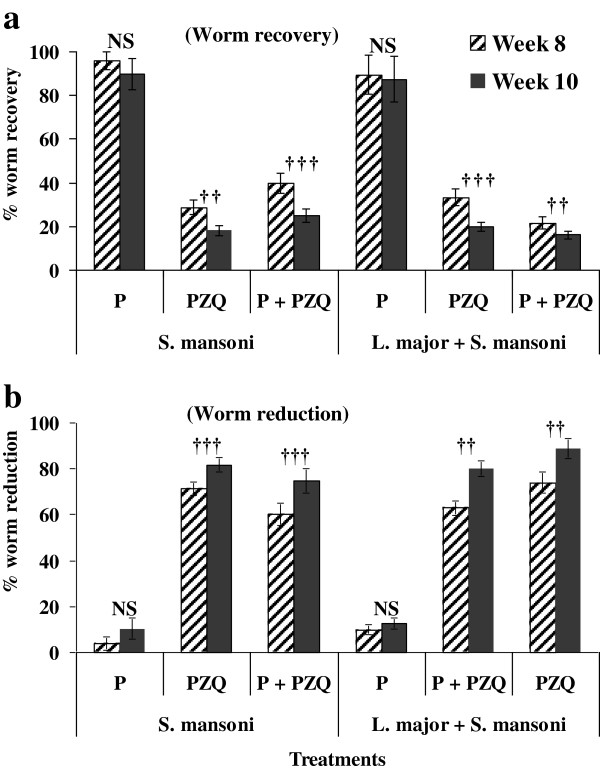
**Worm recovery and reduction in BALB/c mice infected with *S. mansoni *and those co-infected with *L. major *and *S. mansoni *under different treatments.** Symbol †, †† and ††† significant differences in worm reduction/recovery at ∝ = 0.05, 0.01 and 0.001 respectively using Tukey’s HSD Test. NS denotes no significant differences among treatments.

In the *S. mansoni* and *L. major* + *S. mansoni* infected BALB/c mice, there was a significant (*p* < 0.05) reduction in the% worm recovery when PZQ and P + PZQ was applied in managing the infection albeit there was no significant (*p* > 0.05) difference observed when Pentostam was used (Figure 3a). Subsequently, % worm reduction increased when PZQ and P + PZQ was administered in the treatment (Figure 3b).

## Discussion

The drugs recommended in the management of *Leishmania* are pentavalent antimonials such as Pentostam, while management of *Schistosoma* is done using PZQ. Yet, the interactions among parasitic agents in the hosts may alter the epidemiology, development and severity of the disease [1] with unknown consequences on the efficacy of the combined first line treatments. The aim of the present study was to evaluate the efficacy of combined first line of treatment in managing co-infection of *L. major* and *S. mansoni* in BALB/c mice. There was no evidence of antagonistic effects of PZQ on Pentostam, or a possible additive and/or synergistic effect on Pentostam during *Leishmania* mono-infection treatment, which may be associated with PZQ and Pentostam distinct targets and mode of action [36-40,57,58]. Studies in murine models indicate concurrent infection of *L. major* and *S. mansoni*, exacerbate lesion size and/or inability to resolve footpad lesions as rapidly as mice infected with *L. major* which correlates with the impaired ability to rescind parasitic burden [22]. Therefore, it is apparent that a strategy to enhance the resolution of infection in mice is through targeting the *S. mansoni*. In this study, when we applied combined chemotherapeutics of first line treatment for *S. mansoni* (PZQ) and *L. major* (Pentostam) comorbidity, there were large reductions of lesion size in the mice compared to the reduction in lesion size in BALB/c treated with Pentostam or PZQ alone. This observation reveals that through action on *S. mansoni*, the Pentostam acts much faster to heal the lesion in BALB/c mice. Previously, pre-infection with *S. mansoni* delayed the resolution of *L. major* lesions while *L. major* infection had no impact on the course of *S. mansoni* infection in co-infected mice [24]. Since the granuloma response by *S. mansoni* forms a discrete niche that facilitates the intracellular survival of *Leishmania*[26], the reduction of *S. mansoni* through the first line of treatment could enhance the healing response in co-infected mice. It is also probable that healing in combined therapy treatment was achieved through immune response pathways [39,57-60]. Nonetheless, the co-infected BALB/c mice under mono-therapy with Pentostam had significantly smaller lesion nodules as compared to the PZQ treated mice that had no significant difference between the start of treatment and after treatment. Although subject to further scrutiny, it would be possible that the divergence would be explained by having the two arms of immunity operational at the same time. Treatment of *S. mansoni* infection using PZQ has been demonstrated to result in better Th2 responses thus limiting Th1 responses, which tend to abrogate *Leishmania* infection [24,25,34,39,40,58,61-63].

Co-infection may cause an increased stress response in mice manifested through reduced physiological functioning and resulting in reduced growth response [64]. Body weights exhibited growth response to treatments in the current study. The largest increase in body weight occurred in mice infected with *L. major* and treated with Pentostam, and in *S. mansoni* infected mice and treated with PZQ, suggesting efficacy of the first line treatments against mono-parasite infections as previously determined [48,50,60-63]. However, the lower percentage increase in body weight of the *L. major* infected mice after treatment with P + PZQ suggest a more pathological response of BALB/c mice to one of the drugs indicating that combined therapy may tend to be more toxic to mice infected by one parasite than single therapy administration. Although pathological effects of combined Pentostam and PZQ has rarely been shown in co-infected mice, we observed that between week five and six there were signs of toxicity [65] in BALB/c mice co-infected with *L. major* and *S. mansoni* and subjected to concurrent treatment with P + PZQ. In the co-infected mice, the use of combined therapy was found to result in a slight increase in the body weight, which may be concomitant to normal somatic growth. The reduced body weights in BALB/c mice co-infected with *L. major* and *S. mansoni* and treated with either Pentostam or PZQ therapies alone suggest that elimination of one parasite by the single drug does not preclude the pathological impacts of the other parasites resulting in physiological impairment of the mice and hence the observed reduced body weight.

Higher splenomegaly has been previously associated with high parasite burden in co-infected conspecifics [22]. Naturally therefore, the elimination of the parasite tends to reverse the condition. The reduction in the weight of spleen in BALB/c infected with *L. major* and *S. mansoni* and treated with Pentostam and PZQ respectively confirm the efficacy of the first line treatment drugs on these two parasites, which appeared to reduce splenomegaly. However, the non-significant increase in the weight of the spleen between week 8 to 10 suggested efficacious control of the parasites and the increased weight of the spleen was a response to normal somatic growth.

In diverse pathogenesis and pathology findings, infection with the parasitic helminth *S. mansoni* is predominantly associated with liver enlargement resulting in hepatomegaly, which although less severe, has been shown in the presence of *L. major* infection in murine models and hence possible aggravated hepatomegaly in concomitance of the two parasitic diseases [22,66-70]. In the present study, chemotherapy with first line treatments for *L. major* or *S. mansoni* single infection reduced the liver weights between week 8 and 10 suggesting reduced incidence of hepatomegaly which concurs with previous findings [50,71]. The increased liver weight suggests higher incidences of hepatomegaly in the co-infected mice when monotherapy was used as treatment modality. Indicating that modulation of hepatomegaly requires the elimination of both parasites from the BALB/c mice. We confirmed in the present study that when combined therapy of P + PZQ was applied during co-infection, there were non-significant increases in the liver weights between week 8 and 10 pointing to elimination of the parasites that may cause hepatomegaly.

The study also established that in BALB/c mice co-infected with *L. major* + *S. mansoni*, there was a significant reduction in LDU and *S. mansoni* worm counts between week 8 and 10 when treatment was carried out using P + PZQ. The results concur with previous findings that demonstrated respective chemotherapy for the helminth or protozoan parasites at curative doses effectively reducing parasitic loads; even in the absence of total elimination of the parasites at curative or subcurative dosages particularly in parasitic helminth infection, the need for repeated chemotherapy to attain optimal therapy is considered to be core [41,49,50,69,72-74]. However, when combination therapy was used during the co-infection, signs of toxicity were noted at the start of the treatment, perhaps due to accumulation of the drugs in the tissues, particularly in liver and spleen and associated Pentostam reversible cardio toxicity among other side effects of PZQ and Pentostam previously reported [37-40,75]. The reduction of parasitic load in the current study in the combined therapy may reflect the mode of action of the drugs. Pentostam acts upon several targets that include influencing the bioenergetics of *Leishmania* parasite by inhibiting parasite glycolysis, fatty acid beta-oxidation and inhibition of ADP phosphorylation [36,37,57]. Praziquantel produces a well-documented effect on intracellular Ca^2+^ levels in adult schistosomes; within 5 minutes of exposure to the drug, adult schistosomes exhibit a rapid sustained contraction of the worm’s musculature and vacuolization and disruption of the parasite tegument, an effect associated with the subsequent exposure of parasite antigens on the surface of the worm [58].

For a long time, treatment of parasite infections in murine models has focused on the single therapy treatments which use first line treatment that is generally acceptable. Recent cases of increased incidences of polyparasitism in vertebrates call for better ways to manage co-infections. These not withstanding, interactions between co-infecting parasites that have pronounced consequences for disease development, severity and transmission dynamics may complicate the management of concomitance. The results of the present study demonstrate that the use of combined therapy resulted in management of *Leishmania* and *Schistosoma* in BALB/c mice.

## Conclusions

In the present study, treatment of BALB/c mice co-infected with *L. major* and *S. mansoni* was achieved through combination therapy using first line treatments of Pentostam and Praziquantel, representing the first line treatments of *Leishmania* and *Schistosoma* respectively. Combined therapy resulted in reduced lesion size, low parasite burden and appeared to have fewer effects on the physiology of the BALB/c mice. Although most of the parasitic infections are managed as single infections, the problem of co-infection among patients is increasingly being recognized. The results obtained demonstrate that management of co-infections can be achieved by appropriate application of combined therapy of first line treatment, which will go a long way in the management of some of the most neglected tropical diseases.

## Competing interests

The authors declare that they have no competing interests.

## Authors’ contributions

This work was carried out in collaboration between all authors. CKW, VCSN, COA and HLM conceived and designed the study. CKW, JI, LWK, KSG and BJ performed the experiments. COA, CKW, JI and O-OE contributed reagents/materials/analysis tools and logistical support. CKW, O-OE and COA analyzed the data. All the authors participated in drafting and revising the manuscript. All authors read and approved the final manuscript.

## References

[B1] Petney TN Andrews RH Multiparasite communities in animals and humans: frequency, structure and pathogenic significance Int J Parasitol 1998 28 377 393 10.1016/S0020-7519(97)00189-6 9559357

[B2] Cox FEG Concomitant infections, parasites and immune responses Parasitol 2001 122 23 38 10.1017/S003118200001698X 11442193

[B3] Raso G Luginbhuhl A Adjoua CA Tian-Bi NT Silue KD Matthys B Vounatsou P Wang Y Dumas ME Holmes E Singer BH Tanner M N’goran EK Utzinger J Multiple parasite infections and their relationship to self-reported morbidity in a community of rural Cote d’Ivoire Int J Epidemiol 2004 33 1092 1102 10.1093/ije/dyh241 15256525

[B4] Utzinger J Becker SL Knopp S Blum J Neumayr AL Keiser J Hatz CF Neglected tropical diseases: diagnosis, clinical management, treatment and control Swiss Med Wkly 2012 142 w13727 23180107 10.4414/smw.2012.13727

[B5] Ezenwa VO Etienne RS Luikart G Beja-Pereira A Jolles AE Hidden consequences of living in a wormy world: nematode-induced immune suppression facilitates tuberculosis invasion in African buffalo Am Nat 2010 176 613 624 10.1086/656496 20849271

[B6] Telfer S Lambin X Birtles R Beldomenico P Burthe S Paterson S Begon M Species interactions in a parasite community drive infection risk in a Wildlife Population Sci 2010 330 243 246 10.1126/science.1190333 PMC3033556 20929776

[B7] Singh N Kumar M Singh RK Leishmaniasis: current status of available drugs and new potential drug targets Asian Pac J Trop Med 2012 5 485 497 10.1016/S1995-7645(12)60084-4 22575984

[B8] Alvar J Vélez ID Bern C Herrero M Desjeux P Cano J den Boer JM The WHO Leishmaniasis Control Team Leishmaniasis worldwide and global estimates of its incidence PLoS One 2012 7 e35671 10.1371/journal.pone.0035671 22693548 PMC3365071

[B9] Chitsulo L Engels D Montresor A Savioli L The global status of schistosomiasis and its control Acta Trop 2000 77 41 51 10.1016/S0001-706X(00)00122-4 10996119 PMC5633072

[B10] Gryseels B Polman K Clerinx J Kestens L Human schistosomiasis Lancet 2006 368 1106 1118 10.1016/S0140-6736(06)69440-3 16997665

[B11] Hotez PJ Kamath A Neglected tropical diseases in sub-Saharan Africa: review of their prevalence, distribution, and disease burden PLoS Negl Trop Dis 2009 3 e412 10.1371/journal.pntd.0000412 19707588 PMC2727001

[B12] Gryseels B Schistosomiasis Infect Dis Clin North Am 2012 26 383 397 10.1016/j.idc.2012.03.004 22632645

[B13] Odiere MR Rawago F Ombok M Secor WE Karanja DMS Mwinzi PNM Lammie PJ Won K High prevalence of schistosomiasis in Mbita and its adjacent islands of Lake Victoria, Western Kenya Parasit Vectors 2012 5 278 10.1186/1756-3305-5-278 23206383 PMC3523971

[B14] el Gaddal AA The Blue Health Project: a comprehensive approach to prevention and control of water-associated diseases in irrigated schemes of Sudan J Trop Med Hyg 1985 88 47 56 4032529

[B15] Muigai RK Wasunna K Gachihi G Kirigi G Mbugua J Were JBO Schistosomiasis caused by *Schistosoma mansoni* in Baringo district, Kenya—case-report East Afr Med J 1989 66 700 702 2515054

[B16] Zijstra EE Ali AM el-Tourn IA Saitti M Ghalib HW Sondorp E Winkler A Kala-azar in displaced people from Southern Sudan: epidemiological, clinical and therapeutic findings Trans R Soc Trop Med Hyg 1991 85 365 369 10.1016/0035-9203(91)90293-8 1658990

[B17] Hotez PJ Molyneux DH Fenwick A Ottesen E Ehrlich Sachs S Sachs JD Incorporating a rapid-impact package for neglected tropical diseases with programs for HIV/AIDS, tuberculosis, and malaria PLoS Med 2006 3 e102 10.1371/journal.pmed.0030102 16435908 PMC1351920

[B18] Steinmann P Keiser J Bos R Tanner M Utzinger J Schistosomiasis and water resources development: systematic review, meta-analysis, and estimates of people at risk Lancet Infect Dis 2006 6 411 425 10.1016/S1473-3099(06)70521-7 16790382

[B19] Hotez PJ Molyneux DH Fenwick A Kumaresan J Ehrlich Sachs S Sachs JD Savioli L Control of neglected tropical diseases N Engl J Med 2007 357 1018 1027 10.1056/NEJMra064142 17804846

[B20] O’Neal SE Guimaraes LH Machado PR Alcantara L Morgan DJ Passos S Influence of helminth infections on the clinical course of and immune response to Leishmania braziliensis cutaneous leishmaniasis J Infect Dis 2007 195 142 148 10.1086/509808 17152018

[B21] MT G á-P Osuna A Chagas disease in a wormy world Rev Ibero-Latinoam Parasitol 2012 71 Suppl1 5 13

[B22] Yole DS Shamala KT Kithome K Gicheru MM Studies on the interaction of *Schistosoma mansoni and Leishmania major* in experimentally infected BALB/c mice Afr J Health Sci 2007 14 80 85

[B23] Yoshida A Maruyama H Yabu Y Amano T Kobayakawa T Ohta N Immune responses against protozoal and nematodal infection in mice with underlying *Schistosoma mansoni* infection Parasitol Int 1999 48 73 79 10.1016/S1383-5769(99)00006-9 11269328

[B24] La Flamme AC Scott P Pearce E Schistosomiasis delays lesion resolution during *Leishmania major* infection by imparing parasite killing by macrophages Parasite Immunol 2002 24 339 345 10.1046/j.1365-3024.2002.00473.x 12164819

[B25] Hassan MF Zhang Y Engwerda CR Kaye MP Sharp H Bickle QD The *Schistosoma mansoni* hepatic egg granuloma provides a favorable microenvironment for sustained growth of *Leishmania donovani* Am J Pathol 2006 169 Suppl 3 943 953 16936268 10.2353/ajpath.2006.051319 PMC1698825

[B26] Abruzzi A Fried B Coinfection of Schistosoma (Trematoda) with bacteria, protozoa and helminths Adv Parasitol 2011 77 1 85 22137582 10.1016/B978-0-12-391429-3.00005-8

[B27] Buck AA Anderson RI MacRae AA Epidemiology of poly-parasitism. IV. Combined effects on the state of health Tropenmed Parasitol 1978 29 253 268 726041

[B28] King CH Dickman K Tisch DJ Reassessment of the cost of chronic helminthic infection: a meta-analysis of disability-related outcomes in endemic schistosomiasis Lancet 2005 365 1561 1569 10.1016/S0140-6736(05)66457-4 15866310

[B29] Mosmann TR Sad S The expanding universe of T-cell subsets: Th1, Th2 and more Immunol Today 1996 17 138 146 10.1016/0167-5699(96)80606-2 8820272

[B30] Jankovic D Sher A Segel LA, Cohen IR Th1/Th2 effector choice in the immune system: a developmental program influenced by cytokine signals Design principles for the immune system and other distributed autonomous systems 2001 New York: Oxford University Press 79 93

[B31] Maizels RM Yazdanbakhsh M Immune regulation by helminth parasites: cellular and molecular mechanisms Nat Rev Immunol 2003 3 733 744 10.1038/nri1183 12949497

[B32] Hartgers FC Yazdanbakhsh M Co-infection of helminths and malaria: modulation of the immune responses to malaria Parasite Immunol 2006 28 497 506 10.1111/j.1365-3024.2006.00901.x 16965285

[B33] Specht S Hoerauf A Does helminth elimination promote or prevent malaria? Lancet 2007 369 446 447 10.1016/S0140-6736(07)60210-4 17292747

[B34] McManus DP Loukas A Current status of vaccines for schistosomiasis Clin Microbiol Rev 2008 21 225 242 10.1128/CMR.00046-07 18202444 PMC2223839

[B35] Osada Y Kanazawa T Schistosome: its benefit and harm in patients suffering from concomitant diseases J Biomed Biotechnol 2011 2011 ID 264173 10.1155/2011/264173 PMC3216407 22131800

[B36] Croft SL Yardley V Chemotherapy of Leishmaniasis Curr Pharm Des 2002 8 319 342 10.2174/1381612023396258 11860369

[B37] Croft SL Coombs GH Leishmaniasis-current chemotherapy and recent advances in the search for novel drugs Trends in Parasitol 2003 19 Suppl 11 502 508 10.1016/j.pt.2003.09.008 14580961

[B38] Roy P Das S Auddy RG Mukherjee A Biological targeting drug delivery in control of Leishmaniasis J Cell Anim Biol 2012 6 73 87

[B39] Utzinger J Keiser J Shuhua X Tanner M Singer BH Combination Chemotherapy of Schistosomiasis in Laboratory Studies and Clinical Trials Antimicrob Agents Chemother 2003 47 Suppl 5 1487 1495 12709312 10.1128/AAC.47.5.1487-1495.2003 PMC153321

[B40] Ali BH A short review of some pharmacological, therapeutic and toxicological properties of Praziquantel in man and animals Pak J Pharm Sci 2006 19 Suppl 2 170 175 16751131

[B41] Molyneux DH Hotez PJ Fenwick A “Rapid-impact interventions”: How a policy of integrated control for Africa’s neglected tropical diseases could benefit the poor PLoS Med 2005 2 11 e336 10.1371/journal.pmed.0020336 16212468 PMC1253619

[B42] Utzinger J Raso G Brooker S de Savigny D Tanner M Ørnbjerg N Singer BH N’Goran EK Schistosomiasis and neglected tropical diseases: towards integrated and sustainable control and a word of caution Parasitol 2009 136 1859 1874 10.1017/S0031182009991600 PMC2791839 19906318

[B43] Sundar S Rai M Chakravarty J Agarwal D Agrawal N Vaillant M Olliaro P Murray HW New treatment approach in Indian visceral leishmaniasis: Single-dose liposomal amphotericin B followed by short-course oral miltefosine Clin Infect Dis J 2008 47 1000 1006 10.1086/591972 18781879

[B44] Smithers SR Terry RJ The infection of laboratory hosts with the cercariae of *Schistosoma mansoni* and recovery of adults worms Parasitol 1965 5 695 700 10.1017/s0031182000086248 4957633

[B45] Green CJ Animal anesthesia. Laboratory Animal Handbooks 8 1979 London UK: Laboratory Animals Ltd

[B46] Hendricks LD Wright N Diagnosis of Cutaneous Leishmaniasis by *in vitro* cultivation of saline aspirates in *Schneider’s Drosophila* Medium Am J Trop Med Hyg 1979 28 962 964 507285 10.4269/ajtmh.1979.28.962

[B47] Kimber CD Evans DA Robinson BL Peters W Control of yeast contamination with 5-fluorocytosine in the *in vitro* cultivation of *Leishmania spp* Am J Trop Med Parasitol 1981 75 453 454 10.1080/00034983.1981.11687463 7030240

[B48] Wabwoba BW Anjili CO Ngeiywa MM Ngure PK Kigondu EM Ingonga J Makwali J Experimental chemotherapy with *Allium sativum* (Liliaceae) methanolic extracts in rodents infected with *Leishmania major* and *Leishmania donovani* J Vector Borne Dis 2010 47 160 167 20834086

[B49] Fallon PG Doenhoff MJ Drug-resistant schistosomiasis: Resistance to Praziquantel and Oxamniquine induced in *Schistosoma mansoni* in mice is drug specific Am J Trop Med Hyg 1994 51 83 88 8059919 10.4269/ajtmh.1994.51.83

[B50] Chaiworaporn R Maneerat Y Rojekittikhun W Ramasoota P Janecharut T Matsuda H Kitikoon V Therapeutic effect of subcurative dose Praziquantel on *Schistosoma mansoni* infected mice and resistance to challenge infection after treatment Southeast Asian J Trop Med Public Health 2005 36 Suppl 4 846 852 16295535

[B51] Nolan TJ Farrel JP Experimental Infection of the Multimammate Rat (*Mastomys Natalensis*) with *L. Donovani* and *L. major* Am J Trop Med Hyg 1987 36 Suppl 2 264 269 3826485 10.4269/ajtmh.1987.36.264

[B52] Doenhoff M Bickle Q Long E Bain J McGregor A Factors affecting the acquisition of resistance against Schistosoma mansoni in the mouse. I. Demonstration of resistance to reinfection using a model system that involves perfusion of mice within three weeks of challenge J Helminthol 1978 52 173 186 10.1017/S0022149X00005344 722037

[B53] Bradley DJ Kirkley J Regulation of *Leishmania* populations within the host: the variable course of *Leishmania donovani* infections in mice Clin Exp Immunol 1977 30 119 129 606433 PMC1541173

[B54] Engwerda CR Murphy ML Cotterell SE Smelt SC Kaye PM Neutralization of IL-12 demonstrates the existence of discrete organspecific phases in the control of Leishmania donovani Eur J Immunol 1998 28 669 680 10.1002/(SICI)1521-4141(199802)28:02<669::AID-IMMU669>3.0.CO;2-N 9521077

[B55] Zar JH Biostatistical Analysis 1999 4 Englewood Cliffs, New Jersey, USA: Prentice Hall

[B56] Michael K Douglas M Design and Analysis of Experiments 2004 6 Hoboken, USA: John Wiley & Sons, inc

[B57] Chakraborty AK Majumder HK Mode of action of Pentavalent antimonials: specific inhibition of type I DNA topoisomerase of *Leishmania donovani* Biochem Biophys Res Commun 1988 152 605 611 10.1016/S0006-291X(88)80081-0 2835038

[B58] Greenberg RM Are Ca^2+^channels targets of Praziquantel action? Int J Parasitol 2005 35 1 9 10.1016/j.ijpara.2004.09.004 15619510

[B59] Fallon PG Cooper RO Probert AJ Doenhoff MJ Immune-dependent chemotherapy of schistosomiasis Parasitol 1992 105 41 48 10.1017/S003118200007534X 1308928

[B60] Monzote L Current treatment of leishmaniasis: a review The Open Antimicrob Agnts J 2009 1 9 19

[B61] Doenhoff MJ Sabah AA Fletcher C Webbe G Bain J Evidence for an immune-dependent action of Praziquantel on *Schistosoma mansoni* in mice Trans R Soc Trop Med Hyg 1987 81 947 951 10.1016/0035-9203(87)90360-9 3140436

[B62] Chensue SW Warmington KS Ruth J Lincoln PM Kunkel SL Cross-regulatory role of interferon-gamma (IFN-γ), IL-4 and IL-10 in schistosome egg granuloma formation: in vivo regulation of Th activity and inflammation Clin Exp Immunol 1994 98 395 400 7994903 10.1111/j.1365-2249.1994.tb05503.x PMC1534488

[B63] Shuhua X Binggui S Chollet J Tanner M Tegumental changes in adult *Schistosoma mansoni* harboured in mice treated with Praziquantel enantiomers Acta Trop 2000 76 107 117 10.1016/S0001-706X(00)00076-0 10936569

[B64] Retana-Márquez S Bonilla-Jaime H Vázquez-Palacios G Domi’nquez-Salazar E Marti’nez-Garcia R Velázguez-Moctezuma J Body weight gain and diurnal differences of corticosterone changes in response to acute and chronic stress in rats Psychoneuroendocrinol 2003 28 207 227 10.1016/S0306-4530(02)00017-3 12510013

[B65] Organization for Economic Co-operation and Development (OECD) Test Guideline 423. Acute Oral Toxicity – Acute Toxic Class Method 2001 2 rue André-Pascal 75775 Paris Cedex 16 France: OECD

[B66] Doenhoff M Musallam R Bain J Mcgregor A *Schistosoma mansoni* infections in T-cell deprived mice, and the ameliorating effect of administering homologous chronic infection serum: I. Pathogenesis Am I Trop Med Hyg 1979 28 Suppl 2 260 273 10.4269/ajtmh.1979.28.260 313161

[B67] Byram JE Doenhoff MJ Musallam R Brink LH Vonlichtenberg F *Schistosoma mansoni* infections in T-cell deprived mice, and the ameliorating effect of administering homologous chronic infection serum: II. Pathology Am I Trap Med Hyg 1979 28 Suppl 2 274 285 10.4269/ajtmh.1979.28.274 313162

[B68] King CL Mahmond AAF Initiation and regulation of disease in schistosomiasis, Volume 3 2001 London: Imperial College Pres 115 132

[B69] Ross AG Bartley PB Sleigh AC Olds GR Li Y Williams GM McManus DP Schistosomiasis N Engl J Med 2002 346 1212 1220 10.1056/NEJMra012396 11961151

[B70] Bindseli E Iburg T Hurst MH Johansen MV Distinguish periportal fibrosis from portal fibrosis in hepatic Schistosomiasis Trends Parasitol 2004 29 361 362 10.1016/j.pt.2004.05.009 15246318

[B71] Abdul-Ghani RA Loutfy N Hassan A Experimentally promising antischistosomal drugs: a review of some drug candidates not reaching the clinical use Parasitol Res 2009 105 899 906 10.1007/s00436-009-1546-2 19588166

[B72] Fallon PG Hamiliton JV Doenhoeff MJ Efficacy of treatment murine *Schistosoma mansoni* infection with Praziquantel and Oxamniquine correlates with infection intensity: role of host antibody Parasitol 1995 111 59 66 10.1017/S003118200006460X 7609991

[B73] Sabah AA Fletcher C Webbe G Doenhoff MJ *Schistosoma mansoni*: chemotherapy of infections of different ages Exp Parasitol 1986 61 Suppl 3 294 303 3086114 10.1016/0014-4894(86)90184-0

[B74] Makwali JA Wanjala FME Kiburi JC Ingonga J Wabwoba WB Anjili CO Combination and monotherapy of *Leishmania major* infection in BALB/c mice using plant extracts and herbicides J Vector Borne Dis 2012 49 1 8 23135005

[B75] Croft SL Sundar S Fairlamb AH Drug resistance in Leishmaniasis Clin Microbiol Rev 2006 19 Suppl 1 111 126 16418526 10.1128/CMR.19.1.111-126.2006 PMC1360270

